# Interference Field Control for High-Uniformity Nanopatterning: A Review

**DOI:** 10.3390/s25185719

**Published:** 2025-09-13

**Authors:** Jingwen Li, Xinghui Li

**Affiliations:** Shenzhen International Graduate School, Tsinghua University, Shenzhen 518071, China; lijw23@mails.tsinghua.edu.cn

**Keywords:** interference lithography, wavefront engineering, polarization modulation, fringe locking, periodic structures, nanopatterning uniformity

## Abstract

Interference lithography (IL) offers high throughput, excellent uniformity, and maskless patterning capabilities. Compared to other methods, IL enables large-area, cost-effective fabrication of periodic structures with subwavelength resolution, which is particularly valuable for sensing applications, enabling the development of more sensitive, high-resolution, and reliable sensors. This review provides a comprehensive analysis of IL from the perspective of optical field control. We first introduce the principles of interference field formation and summarize key system architectures, including Mach–Zehnder and Lloyd’s mirror configurations, as well as advanced schemes such as multi-beam interference and multi-step exposure for complex pattern generation. We then examine how wavefront engineering, polarization modulation, and phase stabilization influence pattern morphology, contrast, and large-area uniformity. To address dynamic drifts caused by environmental perturbations, both passive vibration isolation and active fringe-locking techniques are discussed. For fringe-locking systems, we review methods for drift monitoring, control algorithms, and feedback implementation. These developments enhance the capability of IL systems to deliver nanoscale accuracy under dynamic conditions, which is essential for stable and high-performance sensing. Looking ahead, IL is evolving into a versatile platform for sensor-oriented nanofabrication. By integrating physical modeling, precision optics, and real-time control, IL provides a robust foundation for advancing next-generation sensing technologies with higher sensitivity, resolution, and reliability.

## 1. Introduction

**Figure 1:**
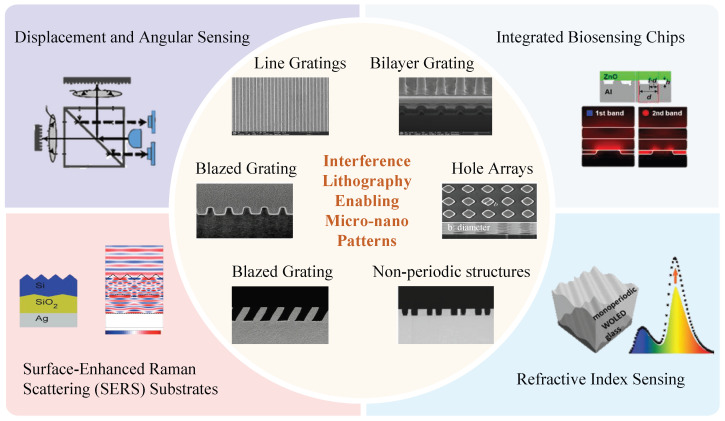
Fabricated structures and applications of interference lithography.

Interference lithography, owing to its high throughput, excellent uniformity, and maskless processing capabilities, has become a pivotal technique for the large-area fabrication of periodic micro/nanostructures [1,2,3]. As the Figure 1 illustrates, beyond its well-established roles in photonic devices [4,5], functional thin films [6], and energy materials [7], it has gained particular prominence in sensing applications [8,9,10]. Periodic nanostructures provide powerful means to modulate electromagnetic fields, enhance light–matter interaction, and generate high-quality resonances, which are indispensable for achieving superior sensing performance [11]. In precision metrology, the integration of interference-lithography-fabricated gratings with interferometric detection schemes enables displacement and angular sensing with nanometer or even sub-nanometer resolution [12,13,14], meeting the stringent demands of ultraprecision manufacturing and positioning systems [15,16,17,18,19]. In optical and biochemical sensing, subwavelength periodic gratings allow precise tailoring of optical coupling, filtering, and dispersion [20,21,22,23], thereby facilitating the development of refractive-index sensors, surface-enhanced Raman substrates, and compact integrated biosensing chips [24,25,26,27]. Periodic arrays with plasmonic or photonic crystal effects further amplify sensitivity by supporting sharp resonance features and localized field enhancement, crucial for label-free detection of trace analytes [28,29,30,31]. Collectively, interference lithography enables scalable and cost-effective fabrication of periodic sensing platforms with high reproducibility, establishing itself as a cornerstone technology for next-generation sensors and metrology devices [3,32,33].

In addition to interference lithography, the fabrication of periodic micro- and nanostructures can also be achieved through a variety of advanced micro/nanomanufacturing techniques, including maskless direct writing, mask transfer, and self-assembly-based patterning approaches [34,35]. Electron-beam lithography (EBL) offers exceptional resolution and is well-suited for the development of high-precision pattern prototypes and mask fabrication [36]; however, its low writing speed and high system cost limit its applicability to large-area production [37,38,39,40]. Laser direct writing (DLW) offers a cost-effective alternative to electron beam lithography (EBL) for high-resolution patterning [41,42,43]; however, its resolution is intrinsically limited by the diffraction of light. To overcome this constraint, two-photon polymerization techniques have been employed, enabling sub-diffraction-limited feature sizes through nonlinear absorption mechanisms [44,45,46,47]. Nanoimprint lithography combines high pattern fidelity with promising scalability, making it ideal for applications requiring structural repeatability, though challenges remain in mold durability and pattern flexibility. Photolithography is fast and widely used in large-scale manufacturing, especially in semiconductor production. It is reliable and well-developed. However, its resolution is limited by the wavelength of light, making it hard to create very small patterns without using complex techniques [48,49,50]. Focused ion beam (FIB) direct writing provides high flexibility and is well-suited for rapid prototyping of microstructures, but it suffers from limited throughput [51,52]. Self-assembly-based techniques such as nanosphere lithography are attractive for their low-cost equipment and process simplicity, and they are applicable in the formation of preliminary patterns [53]. Nonetheless, issues related to pattern controllability and uniformity hinder their use in precision manufacturing [54,55,56].

Compared with these approaches, IL offers a well-balanced combination of scalability, throughput, surface compatibility, and cost-effectiveness for the fabrication of highly ordered periodic nanostructures [57]. A particularly meaningful contrast can be drawn with other maskless, non-lithographic nanostructuring techniques. Direct laser interference patterning (DLIP), which shares the interference principle with IL, enables meter-scale surface processing with roll-to-roll compatibility by directly modifying materials with high-energy laser beams. Nevertheless, its flexibility in generating non-periodic or arbitrary patterns is limited. Laser-induced periodic surface structures (LIPSSs) exploit plasmonic interactions and self-organization effects to spontaneously form sub-wavelength features on diverse materials, but the inherent stochasticity of the process restricts precise control over periodicity and pattern geometry [58,59,60,61]. Nanoimprint lithography (NIL), by contrast, provides excellent replication fidelity and sub-10 nm resolution, yet its strong dependence on mold quality and durability imposes additional costs and constrains design flexibility [62]. Overall, while DLIP highlights scalability, LIPSS expands material compatibility, and NIL advances resolution and pattern fidelity, IL remains a uniquely advantageous technique for efficient, maskless, and large-area production of periodic micro- and nanostructures. A comprehensive evaluation of various fabrication methods is summarized in the Appendix A.

Overall, while each technique has its own performance merits, interference lithography continues to exhibit a compelling advantage due to its maskless operation, efficient generation of highly regular structures, and excellent scalability—making it particularly well-suited for the precise, large-area fabrication of periodic micro- and nanostructures [63,64].

**Figure 2:**
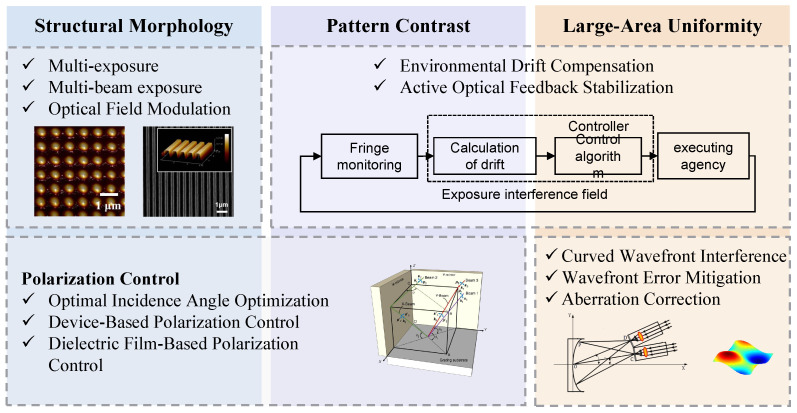
Schematic overview of critical interference field control strategies in nanolithography, including structural morphology modulation, pattern contrast enhancement, large-area uniformity improvement, and polarization/wavefront regulation. These techniques collectively enable high-fidelity, scalable, and tunable nanopatterning.

With the continuous advancement of micro- and nanofabrication technologies, interference lithography has become an increasingly important method for the large-area fabrication of periodic nanostructures [65,66]. Its notable advantages—including high throughput, maskless processing, excellent pattern uniformity, simplified procedures, and low cost—make it a promising option for scalable and efficient nanomanufacturing [67,68,69,70,71]. Interference lithography plays a pivotal role in the fabrication of sensing devices. Its high-throughput capability enables large-area and batch manufacturing [72,73,74], thereby facilitating the development of sensors with broad measurement ranges and high integration [75,76,77]. The simplified processing workflow reduces defects and failure rates, ensuring improved device consistency and reliability [78,79]. By producing highly uniform periodic micro/nanostructures, interference lithography enhances electromagnetic field control and resonance characteristics, which in turn significantly improve sensor sensitivity, linearity, and resolution. These advantages position interference lithography as a key enabling technology for the advancement of next-generation high-performance sensing systems [80,81,82,83]. This review provides a comprehensive overview of the fundamental physical mechanisms and engineering implementations of interference lithography as the Figure 2 shows. It systematically discusses the formation of interference fields, the design and configuration of optical systems, strategies for spatial uniformity modulation, and techniques for maintaining stability of interference fringes. In addition, the current challenges and potential future research directions in the field are critically summarized to guide further advancements in high-throughput and high-precision nanomanufacturing.

## 2. Mechanism of Interference Field Formation and Optical System Configuration

### 2.1. Fundamental Principles of Interference Lithography

**Figure 3:**
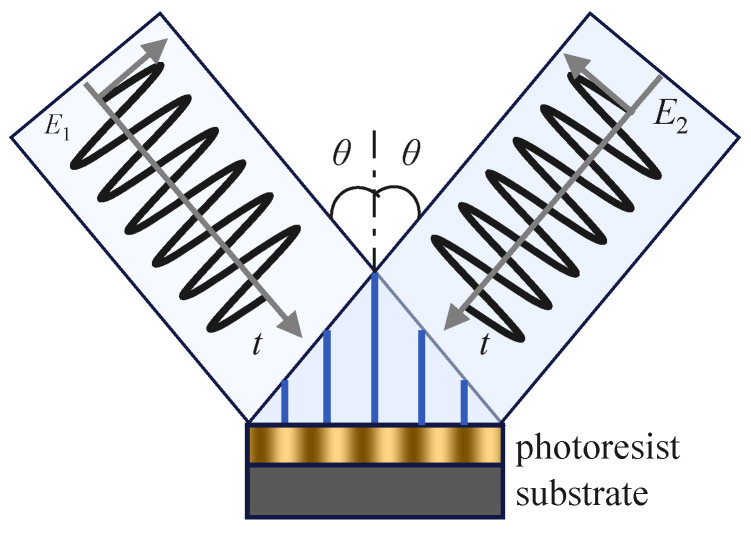
Schematic diagram of interference lithography principle.

Interference lithography (IL) enables high-resolution, maskless pattern replication by forming a stable interference field through the spatial superposition of multiple coherent laser beams [84,85,86]. When directed onto a photoresist-coated substrate, this interference field generates a periodic intensity distribution that defines the resulting micro/nanostructure [87], as the Figure 3 illustrates. In a typical two-beam interference system, the structural period Λ is determined by the wavelength λ and the angle θ between the interfering beams, satisfying the following relation:

(1) $$\Lambda =\frac{\lambda }{2\sin(\theta /2)}$$

For a more general *N*-beam interference configuration, the spatial intensity distribution of the interference pattern can be described as follows:

(2) $$I\left(r\right)=\sum_{i=1}^{N}A_{i}^{2}+2\sum_{m=2}^{N}\sum_{n=1}^{m-1}A_{m}A_{n}\cos\left[\left(k_{m}-k_{n}\right)\cdot r+\psi_{m}-\psi_{n}\right]$$

where $A_{m}$, $k_{m}$, and $\psi_{m}$ represent the amplitude, wavevector, and phase of the *m*-th beam, respectively, and r is the spatial position vector. This formulation reflects that the final pattern arises from the superposition of all pairwise interference terms, with each fringe period $g_{mn}$ determined by the difference in wavevectors between beam pairs. When wavevectors are of equal magnitude and uniformly distributed in a fan-out geometry, the fringe period simplifies to the following:

(3) $$g_{mn}=\frac{\lambda }{2\sin\left(\frac{(m-n)\pi }{N}\right)\sin\theta }$$

In addition, the contrast of the interference fringes is a critical indicator of exposure quality and is defined as follows:

(4) $$C=\frac{I_{\max}-I_{\min}}{I_{\max}+I_{\min}}$$

where $I_{\max}$ and $I_{\min}$ are the maximum and minimum intensities of the interference field, respectively. A high-contrast fringe pattern promotes sharp edges and uniform structural features, which are essential for precise depth control and accurate pattern transfer during subsequent processing steps.

To further overcome the inherent resolution limitations of interference lithography, recent research has extensively explored the use of shorter-wavelength light sources and novel optical mechanisms to enhance exposure performance and enable precise fabrication of finer linewidth patterns. Short-wavelength radiation such as extreme ultraviolet (EUV) and soft X-rays [88,89,90], with wavelengths far below that of visible light, inherently provides superior spatial resolution and has emerged as a key direction in the development of advanced interference lithography systems. By optimizing wavelength selection in conjunction with photoresist chemistry, sub-10 nm periodic structures have been successfully demonstrated [91]. Some schemes have further exhibited the capability to fabricate high aspect ratio features in metallic materials and inclined dielectric substrates. However, such systems typically rely on large-scale synchrotron sources or specialized high-energy equipment. The complexity of system integration and relatively low throughput continue to pose significant challenges for scalable application [92].

Beyond light source optimization, the incorporation of metamaterials and plasmonic structures has offered entirely new approaches for surpassing the diffraction limit. Plasmonic hyperbolic metamaterials (HMMs), such as metal–polymer multilayers with anisotropic hyperbolic dispersion, support the propagation of high spatial frequency modes, enabling resolution far beyond λ/10 even under conventional ultraviolet illumination (e.g., 365 nm). These approaches combine cost-effective implementation with large-area fabrication capabilities, providing a feasible technological pathway for advancing interference lithography toward the 10 nm node [93].

In addition, two-photon interference lithography, based on nonlinear optical responses, enables high-contrast, three-dimensional pattern construction by controlling the depth and phase of the relief gratings. This significantly expands the degrees of freedom and precision in complex structure fabrication via interference lithography [94].

In interference lithography, the choice of photoresist critically influences pattern fidelity and downstream functionality. Due to the sinusoidal nature of the exposure field, photoresists must exhibit high resolution and appropriate spectral sensitivity to ensure accurate pattern transfer at the nanoscale. The aspect ratio of the target structures further dictates mechanical robustness and etch resistance, particularly for high-aspect-ratio features. Moreover, IL patterns may serve as etch masks or directly as functional layers, placing additional demands on the optical, electrical, or mechanical properties of the resist [95,96]. As such, photoresist selection in IL must balance resolution, wavelength compatibility, structural geometry, and functional requirements.

To meet the integrated demands of pattern diversity, dimensional uniformity, and process accuracy in next-generation micro/nano device manufacturing, interference lithography must undergo further refinement in light-field modeling, material response mechanisms, and multi-dimensional control strategies [97,98]. Achieving precise design methodologies and system-level optimization in these domains will be essential for its continued advancement [99,100,101].

### 2.2. Typical System Configurations

**Figure 4:**
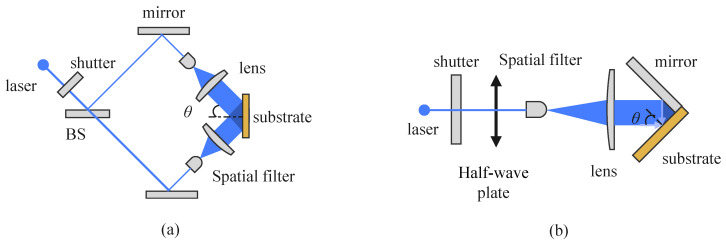
Typical interference lithography system configurations. (**a**) Mach–Zehnder interferometer. (**b**) Lloyd’s mirror interferometer.

The configuration of an interference lithography (IL) system determines the degrees of freedom for optical field control, tunability of fringe periods, and overall system stability. Based on the physical mechanisms underlying interference field formation, IL systems can be broadly categorized into amplitude-splitting and wavefront-splitting types, with the Mach–Zehnder interferometer and Lloyd’s mirror interferometer being the most representative structures, which are illustrate in Figure 4. Some representative works are listed in Table 1.

The Mach–Zehnder interferometer is an amplitude-splitting system in which an incident laser beam is divided by a beamsplitter into two independent paths that are recombined at the sample region to generate interference fringes. This configuration offers high flexibility, allowing independent control of the two beams’ incident angles, polarization states, and phase differences. As such, it enables precise multi-parameter tuning of fringe period, orientation, and contrast, and it is well-suited for constructing complex 2D/3D patterns and implementing multi-beam extensions [105,106,107].

Lloyd’s mirror interferometer, on the other hand, is a compact wavefront-splitting system. It typically consists of a flat mirror placed perpendicular to the sample substrate and an incident light source. A single laser beam illuminates both the sample and the mirror, producing a superposition of direct and reflected beams at the sample surface, which forms periodic fringes. This configuration features simple construction and easy optical alignment. Owing to the shared light source and common-path geometry, it exhibits excellent coherence stability, making it particularly attractive for low-cost, miniaturized IL platforms [85,108,109,110,111].

In recent years, the Lloyd’s mirror configuration has evolved into various modified architectures. Wathuthanthri et al. developed a tunable Lloyd’s interferometer with two degrees of freedom by enabling independent control of the mirror’s rotational angles [104,112]. This design significantly expanded the range of pattern sizes and fringe periods that could be achieved, demonstrating strong potential for applications in nanoscience and large-area pattern fabrication. Other researchers have further optimized the Lloyd’s mirror geometry to reduce periodicity errors or modulate the interference period, enabling the fabrication of chirped microstructures with spatially varying pitch [113,114,115,116]. In addition, Dammann gratings have the capability of generating multiple beams with high uniformity, which makes them suitable for constructing multi-beam interference lithography systems. Dammann gratings have already demonstrated significant applications in parallel laser direct writing [117].

### 2.3. Interference Lithography for Complex Multidimensional Periodic Structures

Interference lithography (IL), owing to its high throughput and large-area patterning capability, has been widely employed for the rapid fabrication of one-dimensional periodic structures. These periodic structures are widely used, especially in precision displacement measurement [118,119,120]. However, in response to the growing demand for two- and three-dimensional periodic architectures in integrated photonics, functional materials, and micro/nanostructured devices, conventional two-beam interference is insufficient to meet the requirements of complex geometries and multiscale structuring.

**Figure 5:**
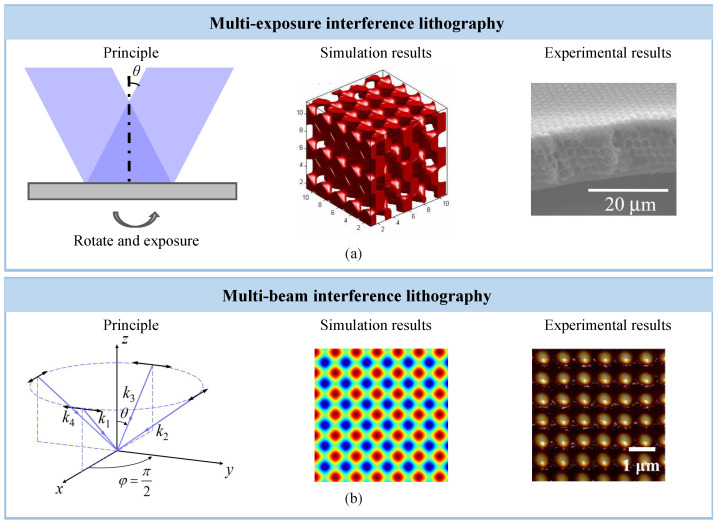
(**a**) Multiple-Exposure method: principle, simulation and experimental results. Reprinted from [94]. (**b**) Multi-beam interference: principle, simulation and experimental results.

To address these challenges, recent research has focused on the spatiotemporal superposition of interference fields, leading to the development of several extended approaches. As the Figure 5 shows, These include multiple-exposure strategies, multi-beam interference techniques, and hybrid configurations that integrate additional physical mechanisms. Such methods have demonstrated great potential in enhancing the dimensionality and complexity of interference-generated patterns, and they represent crucial pathways for advancing IL toward high-dimensional patterning capabilities [121].

#### 2.3.1. Multiple-Exposure Techniques

Multi-exposure interference lithography has emerged as an effective strategy for constructing two- and three-dimensional periodic structures by leveraging angular superposition and dose modulation. This approach enables flexible fabrication of complex morphologies by precisely controlling the sample’s rotation angles, exposure sequences, and the spatial overlap of interference fringes, offering both high adaptability and process controllability [122,123,124].

In typical implementations, multiple rotational exposures allow the superposition of interference fringes from different directions, enabling the realization of diverse spatial configurations such as line gratings, square lattices, and hexagonal close-packed arrays. For instance, Ren et al. developed a multi-exposure method based on guided-mode interference in symmetric metal-clad dielectric waveguides. By selectively exciting distinct waveguide modes (TM and TE) and employing multi-angle exposures, subwavelength patterns with periodicities ranging from 47 to 94 nm were achieved. Notably, three-step rotational exposures enabled the formation of quasi-hexagonal arrays. This technique demonstrated sub-diffraction patterning capabilities under low-cost UV sources and exhibited excellent scalability and compatibility with conventional processes [125,126].

In functional nanostructure fabrication, Chen et al. employed a double-exposure interference lithography scheme to fabricate two-dimensional symmetric gold nanoparticle arrays (2DSGA). They systematically investigated the effect of varying exposure doses on the local electromagnetic field enhancement factor (EF). The results showed that optimal EF distributions were achieved within a total exposure time window of T to 2T, and the resulting nanostructures were insensitive to the polarization angle of incident light. This provides a robust design pathway for high-performance surface-enhanced Raman scattering (SERS) substrates [127,128].

To further enhance large-area pattern uniformity and tunability, Gan et al. proposed a hybrid two-step exposure process that combines interference lithography with grayscale pattern spatial encoding (GPSE). The approach involves initial fabrication of periodic base structures using IL, followed by grayscale exposure to spatially modulate line widths, effectively compensating for pattern distortion induced by laser intensity nonuniformity. This method achieved less than 5% linewidth variation across 4-inch wafers, with a more than 1100% improvement in pattern uniformity, demonstrating exceptional scalability and tunability for large-area nanofabrication [129].

In summary, multi-exposure interference lithography offers a versatile and scalable solution for fabricating complex 2D and quasi-3D micro/nanostructures. Through integration with guided-mode coupling, dose control, and grayscale modulation, this technique shows significant promise in enhancing structural diversity, patterning precision, and functional applicability, representing a key pathway toward scalable manufacturing of complex periodic architectures [130].

#### 2.3.2. Multi-Beam Interference Lithography

Multi-beam interference lithography (MBIL) enables the formation of two-dimensional or quasi-three-dimensional periodic structures by coherently superimposing multiple beams within a single exposure [131,132,133]. Compared to multi-exposure methods, MBIL offers significant advantages in terms of pattern symmetry, fabrication efficiency, and structural complexity. By tuning the incident angles, polarization states, and phase relationships among the beams, the structural period, morphology, and orientation can be flexibly modulated [134,135,136]. This approach has been widely applied in the high-throughput fabrication of micro/nanodevices such as photonic crystals, metamaterials, and functional surfaces [137,138,139].

In recent years, considerable progress has been made in enhancing the structural tunability of multi-beam interference systems. Studies have shown that in six-beam interference configurations, the adjustment of incidence angles enables the generation of patterns with different lattice symmetries, while polarization arrangements (e.g., TE–TE–TE modes) critically affect fringe contrast and imaging quality [140]. Moreover, the integration of MBIL with near-field lithography—using colloidal particle lenses or phase masks—has further improved patterning precision and morphological diversity, enabling controlled fabrication of increasingly complex nanostructures [141]. Precise angular calibration is a critical prerequisite for high-fidelity pattern generation in multi-beam interference lithography. The metrology grating method stands out as a robust technique to achieve this, typically implemented as a two-stage process. The first stage establishes an absolute angular reference by aligning a beam to the Littrow condition with a reference grating, thereby locking its incidence angle to a fundamental physical benchmark defined by the grating period and laser wavelength. The second stage achieves high-sensitivity relative alignment by employing a grating with a period double that of the interference field. This approach leverages the Moiré fringe effect to visually amplify minute, nanoradian-scale angular deviations into macroscopic fringes. Fine-tuning is then performed by adjusting the incident beams to expand these Moiré fringes to an infinite period, signifying near-perfect relative alignment with exceptional precision [142].

Additionally, multi-beam systems have been successfully employed in the batch fabrication of asymmetric nanostructures such as multi-groove and dual-groove arrays. Experimental results demonstrate superior performance in controlling aspect ratios, achieving size uniformity, and ensuring fabrication consistency, highlighting the scalability and engineering potential of MBIL approaches [143].

Overall, multi-beam interference lithography, characterized by its high processing efficiency and precise structural control, is emerging as a powerful tool for generating highly symmetric periodic patterns. It provides strong technological support for the scalable and cost-effective manufacturing of complex micro- and nanostructures over large areas.

#### 2.3.3. Alternative Structure Formation Mechanisms

Beyond conventional strategies such as multi-exposure and multi-beam interference, recent research has introduced a variety of novel physical mechanisms aimed at enhancing the capabilities of interference lithography in terms of structural complexity, dimensional resolution, and pattern diversity [144,145,146].

Yong-Won Ma et al. developed a spiral-path laser exposure system that enables seamless pattern transfer over a rotating cylindrical surface with a length of up to 300 mm. By integrating prism-based beam splitting with high-precision motion control, the system successfully fabricated highly uniform nanoscale patterns with a linewidth of approximately 75 nm and a period of 286 nm, demonstrating strong potential for large-scale roll-to-roll nanoimprint manufacturing [33].

Another representative advancement involves the generation of Bessel beams using a lens–axicon optical system to fabricate concentric circular gratings with tunable periods. Mahyar Mazloumi et al. employed this setup to construct concentric metal rings on azobenzene-doped glass thin films, achieving pattern periods ranging from 469 nm to 660 nm. Surface plasmon resonance (SPR) analysis indicates promising applications in colorimetric sensing and real-time optical imaging [147,148].

Microsphere Self-Interference Lithography (MSIL) presents an effective route for high-resolution patterning without requiring complex optical path control. Zhiwen Gao et al. utilized a self-assembled microsphere array to induce multi-path interference under single-beam laser illumination, enabling the fabrication of nanopore and nanoslit structures with linewidths down to 75 nm and pattern areas exceeding 1 cm^2^ in a single exposure. This method has been applied to the development of high-performance substrates for surface-enhanced Raman scattering (SERS), highlighting its scalability and application potential [149].

Furthermore, Moiré effects have been incorporated into interference lithography systems to enable composite macro–micro structuring and to control the depth of microfeatures, thereby enriching the hierarchical complexity and functional expression of resulting patterns [150,151,152].

Collectively, these emerging approaches significantly broaden the technological boundaries of interference lithography. They not only enable breakthroughs in sub-diffraction-limit patterning but also offer new avenues for multi-scale, high-degree-of-freedom structural design, underscoring the vast development potential of interference lithography in next-generation nanomanufacturing.

## 3. Uniformity Engineering and Structural Modulation in Interference Lithography

### 3.1. Wavefront Adjustment Techniques

In interference lithography, the uniformity of fringe wavefronts directly determines the pattern consistency of periodic structures. This is particularly critical under large-area exposure conditions, where even slight phase aberrations can accumulate, leading to periodicity errors and positional deviations in the resulting structures. For the measurement scenes, it influence the signal quality [153,154]. Therefore, to achieve high spatial uniformity of interference fringes, it is essential to precisely control the wavefront shape and propagation characteristics of the interfering beams. Such control allows for the optimization of the interference field’s uniformity and aberration level at the source, laying the foundation for highly consistent pattern formation [155].

#### 3.1.1. Spherical Wave Interference Errors and Substrate Compensation Strategies

**Figure 6:**
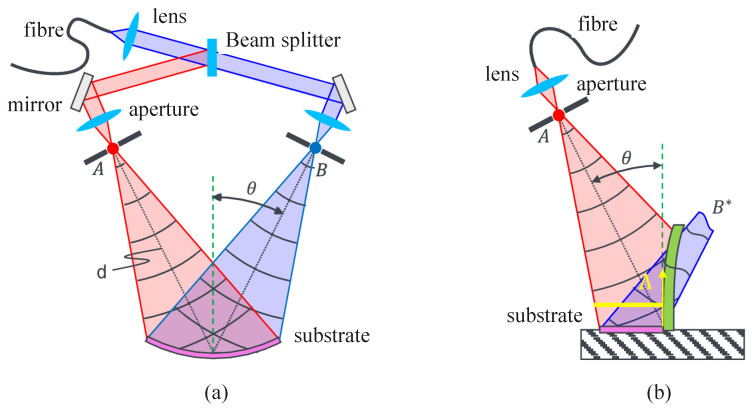
(**a**) Classical LIL setup comprising a Mach–Zehnder interferometer combined with the technique to bend the substrate during the exposure to eliminate the period chirp. (**b**) The Lloyd’s mirror LIL setup. The mirror is deliberately bent to eliminate the period chirp. Reprinted from [120].

In conventional interference lithography systems, two-beam interference typically relies on spherical waves formed through beam expansion and spatial filtering. However, the inherent spatial variation in incidence angles of spherical waves leads to position-dependent fringe periods on the substrate, resulting in nonlinear pattern distortions and limiting the uniformity and precision of large-area fabrication [156,157,158]. As Figure 6 shows, to address this issue, Rao et al. proposed a substrate morphology compensation strategy, wherein a concave vacuum chuck was employed to induce controlled bending deformation of a silicon wafer [159,160]. This deformation compensates for the spatial variation in interference angles. Under a 442 nm laser source, their approach reduced grating period nonuniformity on 4-inch silicon wafers by approximately 86%, with particularly notable improvements in smaller design periods ranging from 500 to 1000 nm, demonstrating strong engineering feasibility and scalability.

It is worth emphasizing that the spatially varying incidence angles inherent to spherical wavefronts can be advantageously exploited in specific applications [161]. For instance, Qian Zhou et al. incorporated a concave lens into a holographic lithography setup to modulate the wavefront shape for the fabrication of large-constant concave gratings, specifically designed for broadband miniature spectrometers [162]. In their configuration, a point light source was passed through the concave lens to generate quasi-spherical waves, thereby simplifying the optical system while maintaining precision. The resulting gratings exhibited a spectral resolution better than 1.1 nm across the 360–825 nm range, demonstrating the distinctive capabilities and promising application potential of spherical wave interference in constructing complex curved micro/nanostructures [163,164,165].

Accurate control of the interference fringe period is also essential for large-area uniformity and for grating interference measurement applications because the critical size is the standard reference [166,167,168,169]. Xiang et al. demonstrated a scanning reference grating (SRG) method enabling in situ period measurement with picometer precision, achieving 833.335 nm ± 10 pm uniformity over 60 mm [170,171]. This approach provides a powerful metrological tool that complements wavefront engineering in advanced holographic lithography.

#### 3.1.2. Aberration Control Strategies of Collimated Beams

To fundamentally reduce the non-uniformity of interference wavefronts, a widely adopted approach involves collimating spherical waves using lenses or mirrors, thereby approximating planar waves within the interference region. Common collimation elements include convex lenses and concave mirrors, among which convex lenses are widely employed due to their simple structure and ease of customization. However, intrinsic lens aberrations (e.g., spherical and coma aberrations) and alignment errors often introduce new wavefront distortions, making precise tuning and compensation essential for system design.

Aspherical lenses, which offer superior aberration control, have been introduced into high-precision systems. In a large-aperture aspherical lens-based interference system developed by a group at Tsinghua University, careful wavefront control reduced the single-exposure grating line position error to below 0.05λ, enabling precise control over cumulative errors after multiple exposures and significantly enhancing the practicality of interference lithography for large-area high-precision structures [172].

Several studies have introduced wavefront shaping devices to enhance the uniformity of energy distribution and duty cycle consistency within the interference region. Dongbai Xue et al. proposed an amplitude-splitting flat-top beam interference lithography (AS-FIL) method, in which a Gaussian beam is converted into a flat-top beam with a diameter of approximately 1 inch and intensity uniformity of 21% using a beam shaper. When the irradiance nonuniformity of the incident beam is kept below 20%, the duty cycle variation of the exposed gratings can be confined within ±2%. This approach enabled the fabrication of uniform photoresist gratings with a line density of 1740 lines/mm over a large area, demonstrating excellent control over pattern homogeneity [173].

Yin-Kuang Yang et al. further developed a flat-top beam shaping device based on tunable air-gap interference. By forming an air cavity between two parallel quartz windows and precisely adjusting the interference layer thickness, the system achieved highly uniform beam outputs. This method delivered a laser utilization rate of 47.4% over a 196 mm^2^ area and significantly increased exposure throughput over a 2000 mm^2^ region, offering an effective strategy to improve the energy efficiency of interference lithography systems [174].

To address systematic alignment errors in collimated optical setups, other studies have employed ray-tracing and Zernike polynomial modeling to analyze aberration mechanisms in spherical lens systems. Results indicate that dominant aberrations originate from relative displacements between dual point sources, and a linear mapping between alignment error and aberration terms has been established to support error compensation strategies. A practical “overlapped grating fringe” method has also been proposed for alignment error diagnosis: by rotating the exposed grating by 180° and inspecting changes in fringe morphology, users are guided to fine-tune the focal position of the lens, enabling error correction on the order of 0.5λ. Despite being limited by visual resolution, this method has been widely adopted in conventional collimation systems.

Building upon these efforts, Wang Shiwei developed a feedback control strategy for dual-beam spherical wave collimation systems based on the extraction of diffraction wave aberrations and Zernike decomposition. By establishing a mapping between the phase distortion of the interference field and the wavefronts of the ±1 diffraction orders and employing ray tracing simulations and wavefront reconstruction, they achieved rapid aberration diagnostics and component-wise correction. Experimental results showed that after two rounds of feedback, the interference aberration was reduced from 0.7λ to 0.03λ, significantly enhancing pattern periodicity and grating stability and offering an efficient, robust pathway for constructing high-uniformity interference lithography systems [175,176].

Precise wavefront shaping is essential for achieving uniform interference fringes and minimizing cumulative pattern errors. However, for the large-size fabrication, the sensing of the wavefront is hard to realize, and it often relies on high-quality aspherical optics or adaptive elements, which increase system cost and alignment complexity [177]. Maintaining stable collimation over large areas further challenges throughput and industrial scalability, as minor misalignments can propagate across exposures.

### 3.2. Polarization Control of Interference Fields

In laser-based fabrication, the polarization state plays a pivotal role in governing the orientation and morphology of surface micro/nanostructures [178]. In interference lithography, the spatial distribution of interference fringes is not only dictated by the configuration of the incident wave vectors but also strongly influenced by the polarization states of the beams. Precise control of the polarization of incident light can effectively enhance fringe contrast, improve pattern uniformity, and enable flexible design of structural symmetry and morphology, thereby becoming a key enabler for the patterning of complex micro- and nanostructures [179,180]. For two-beam interference lithography, which is illustrated in Figure 4a, the optimal configuration is to set both beams to an identical linear polarization state, typically TE-TE (s-polarization), where the electric field vectors are parallel and perpendicular to the plane of incidence, ensuring maximum fringe contrast. In practice, a reference polarization is first defined with a linear polarizer. When a PBS is used for beam splitting, two half-wave plates WP1 and WP2 are introduced: one before the PBS to adjust the splitting ratio, and another to align the polarization of the two beams. The adjustment is guided by the reference polarizer and verified with a power meter to achieve balanced intensity and stable high-contrast interference.

**Figure 7:**
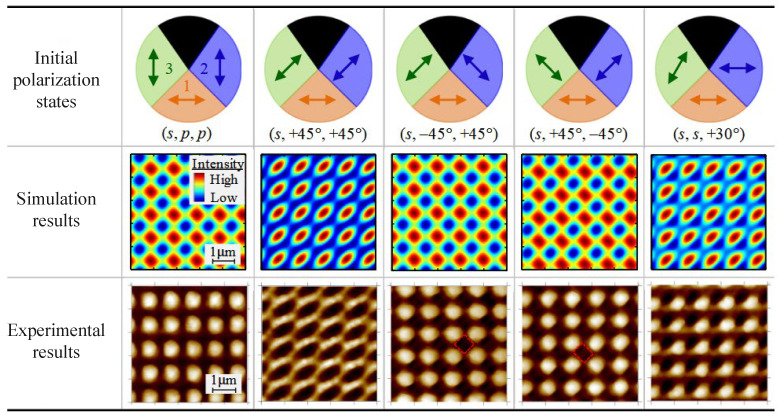
Comparison between the simulated and experimental interference fringes generated by beams 1, 2, and 3 under different combinations of initial polarization states of the three beams. Reprinted from [135]. Arrow directions indicate the respective polarization directions.

In multi-beam interference systems, polarization matching critically influences both fringe orientation and structural morphology. XiuGuo Chen et al. employed a three-dimensional polarized ray-tracing model, combined with half-wave plate tuning, to optimize fringe symmetry and uniformity in a dual-axis Lloyd mirror system for two-dimensional grating fabrication, as Figure 7 shows. Similarly, another study utilized a multi-objective genetic algorithm to optimize the initial polarization states of interfering beams, effectively eliminating cross-interference artifacts caused by single reflections and significantly enhancing the precision and consistency of scale grating fabrication [181,182].

**Figure 8:**
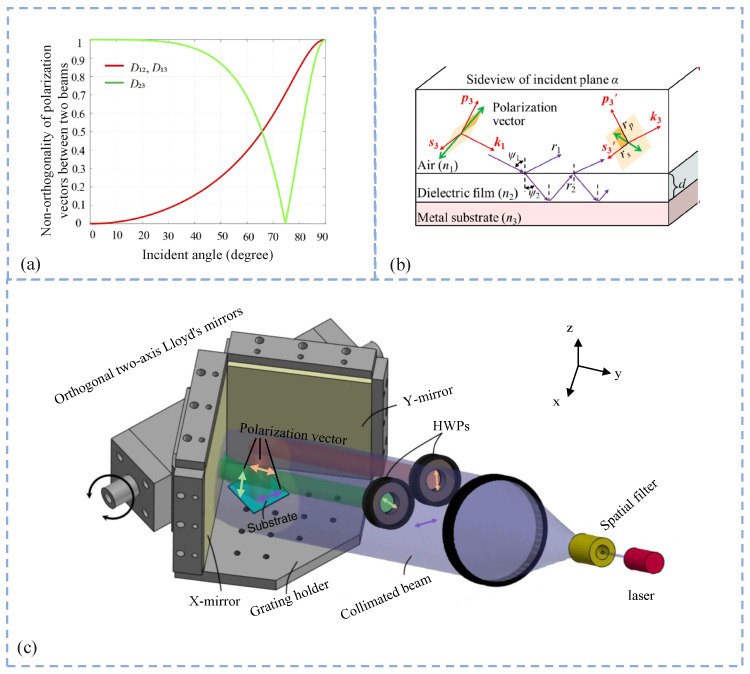
Polarization control strategies in interference lithography systems: (**a**) optimizing the incidence angle; (**b**) applying dielectric coatings on mirror surfaces to manipulate the polarization state upon reflection; (**c**) inserting a half-wave plate to precisely rotate the polarization direction of the beam. Reprinted from [137].

Furthermore, Gaopeng Xue et al. systematically developed a multi-stage polarization control strategy based on a dual-axis Lloyd’s mirror interferometric system, aiming to enhance both the fabrication quality and structural adaptability of two-dimensional (2D) crossed grating structures, which is illustrated in Figure 8. In their earlier study, they constructed a single-beam, single-exposure interferometric system using orthogonal dual-axis Lloyd’s mirrors, and they conducted a comprehensive analysis of the undesired interference caused by non-orthogonal beam overlap. A quantitative index was proposed to evaluate the degree of deviation from orthogonality, enabling the optimization of the incident angle without the need for polarization modulation. Using this approach, highly uniform crossed gratings with periods of approximately 1076/1091nm were successfully fabricated over a large area of $400\;{\mathrm{mm}}^{2}$, achieving a period standard deviation below 0.3% [183]. In subsequent work, they introduced an active polarization control mechanism in which a three-dimensional polarization ray tracing model and tailored initial polarization states were employed to flexibly regulate grating periods within the range of 500–1500 nm while effectively eliminating parasitic interference and enhancing fringe contrast [102]. In their most recent advancement, the team proposed a passive polarization modulation scheme using a MgF_2_ dielectric thin film embedded within the orthogonal Lloyd’s mirror system [184]. This approach enables polarization state tuning at mirror interfaces without requiring additional optical components, thus simplifying the system architecture. As a result, stable exposure of large-area structures ($30\times 30\;{\mathrm{mm}}^{2}$) with tunable periods ranging from 742 to 1500 nm was achieved, delivering a throughput as high as $1.73\times 10^{9}$ [103].

In higher-dimensional systems, J. K. Chua et al. demonstrated controllable pattern transitions from square holes to hexagonal pillars by adjusting polarization directions within a four-beam interference system, underscoring the critical role of polarization in nanostructure morphology tuning.

Recent research efforts have further expanded the implementation of polarization control in three-dimensional light field modulation to meet the demands of complex pattern generation. In the realm of contrast modeling and polarization optimization, Fuping Peng et al. established a simulation framework for polarized fields in a three-beam non-coplanar interference system, revealing the coupled influence of incidence angles and polarization combinations on fringe contrast. They identified that in dual-beam systems, contrast loss is primarily due to polarization mismatch, whereas in three-beam configurations, the TE–TE–TE mode maintains a contrast above 0.97 at large incidence angles, with exceptional tolerance to polarization deviations. These findings provide both theoretical guidance for high-contrast subwavelength patterning and a predictive model for contrast degradation in multi-beam systems [140,185].

To enable higher-precision fabrication, Jiaqi Song et al. proposed a dual-path self-aligned polarized interference lithography (Dp-SAPIL) technique. Through three-dimensional polarized ray-tracing optimization, the system achieves synchronized matching of polarization states and interference paths. Under TE-mode illumination, they demonstrated a highly robust and high-contrast interference field, successfully fabricating rectangular diffraction gratings with a minimum period of 238.3 nm, linewidth of 60 nm, and sidewall angles exceeding $85^{{}^{\circ}}$. Moreover, the system exhibits capability for quasicrystal and projection patterning, paving the way for multifunctional nanostructure manufacturing [186].

In summary, polarization control strategies, through the joint optimization of multidimensional parameters, have significantly expanded the design space and process controllability of interference lithography systems. Particularly when combined with phase modulation, incidence angle tuning, and intelligent optimization algorithms, the spatial control of polarized light fields is evolving towards programmability and high integration.

Polarization management enables enhanced fringe contrast and flexible structural design, but precise control typically requires multi-stage waveplates or dielectric coatings and meticulous calibration. Sensitivity to alignment and environmental fluctuations increases operational complexity, constraining long-term stability and large-area production. Integrated or passive polarization solutions are needed to improve robustness and facilitate industrial implementation.

### 3.3. Spatial Modulation of Interference Fields

By tailoring the periodicity of interference wavefronts, it is possible to introduce controlled deviations from strictly periodic structures, thereby enabling the fabrication of diverse patterns and advanced functional devices. This paradigm shift has extended interference lithography from simple periodic arrays toward quasi-periodic, gradient, and defect-engineered architectures. Among various strategies, spatial light modulators (SLMs) have emerged as a central tool owing to their programmable phase control and high resolution [187]. Behera et al. demonstrated a phase-SLM approach combined with multi-mirror beam steering to realize single-step fabrication of submicron structures including hexagonal lattices, quasicrystals, vortex lattices, and three-dimensional chiral patterns [188]. Lutkenhaus and George reported an electrically addressable SLM-based holographic lithography method, where gray-level modulation enabled precise realization of graded photonic crystals [189]. Ohlinger et al. further combined five-beam interference with SLM control to fabricate gradient-index (GRIN) structures such as Luneburg lenses in a single exposure [190], while Xavier and Joseph developed the defect-engineered multi-plane wave interference (DEMPI) technique, embedding tailored defects into complex lattices through programmable phase control [191]. Collectively, these studies highlight the versatility of SLMs in expanding the design space of interference lithography.

In addition to SLM-based techniques, dynamic phase masks and computational holography have been introduced for frequency modulation. By carefully engineering the phase distribution, these approaches break the intrinsic periodicity and symmetry of conventional interference fields while maintaining process simplicity, offering new opportunities for graded-index optics, complex photonic crystals, and metamaterials.

Spatial modulation of interference beams further enables chirped structures, which provide enhanced design freedom for integrated optics and functional photonics [192]. URA and Kintaka proposed a phase-shift mask-based multi-beam interference approach, successfully fabricating large-area gratings with sawtooth-like chirped distributions and validating its potential for high-chirp-rate grating production [193]. Kim et al. modified the Lloyd’s mirror configuration by incorporating a concave reflector, where tuning the incidence angle of a GaN-based diode laser enabled single-step realization of large-area one- and two-dimensional chirped gratings, confirmed by diffraction and microscopy characterization [194].

Despite these advances, practical challenges remain. Maintaining uniform exposure dose over large areas is essential to suppress structural distortions, while minimizing stitching errors is critical for ensuring pattern continuity and phase coherence. Addressing these challenges—while preserving the design flexibility intrinsic to chirped interference lithography—will be key to its future development.

## 4. Techniques for Spatiotemporal Stabilization of Interference Fringes

### 4.1. Sources and Types of Dynamic Drift in the Interference Field

In interference lithography, the stability of the interference fringes plays a critical role in determining pattern accuracy. However, due to various environmental perturbations, the interference field is prone to dynamic drift, leading to phase shifts, fringe nonuniformity, and pattern distortion. The primary sources of disturbance include thermally induced optical path changes (caused by temperature fluctuations in optical elements or ambient air, which alter the refractive index) [195,196,197], mechanical vibrations (resulting in changes in the optical path difference between interference arms) [198,199], air turbulence (which introduces small perturbations to the beam propagation path) [200], wavelength or power drift of the laser source, and positional or angular changes of the substrate or platform [201]. Depending on the manifestation of interference field variation, fringe drift can be categorized into the following types: phase drift, referring to the translational movement of the entire interference pattern; period drift, which indicates changes in fringe spacing due to variations in the interference angle or laser wavelength; and fringe distortion, which describes the nonlinear deformation of the fringe profile often caused by complex multi-factor interactions.

### 4.2. Passive Stabilization Methods for the Interference Field

Passive stabilization techniques aim to minimize the transmission of external disturbances to the interference field by optimizing the operating environment of the system. Common strategies include:

1. Vibration and acoustic isolation: Employing high-performance optical tables, air-floating vibration isolation systems, and acoustic enclosures to shield the setup from ground vibrations and airborne noise;
2. Temperature and humidity control: Maintaining the experimental environment within ±0.1 °C and ±1% relative humidity using air-conditioning systems and climate control units to reduce thermal expansion and refractive index fluctuations in air;
3. Enclosed beam path design: Utilizing sealed optical paths to prevent air turbulence and particulate contamination, thereby enhancing system stability.

Although passive methods are indispensable in system design, they offer limited suppression of high-frequency random disturbances or small-scale optical path fluctuations within the apparatus. Therefore, active stabilization techniques are required to further enhance system robustness [202].

### 4.3. Active Locking Techniques for the Interference Fields

**Figure 9:**
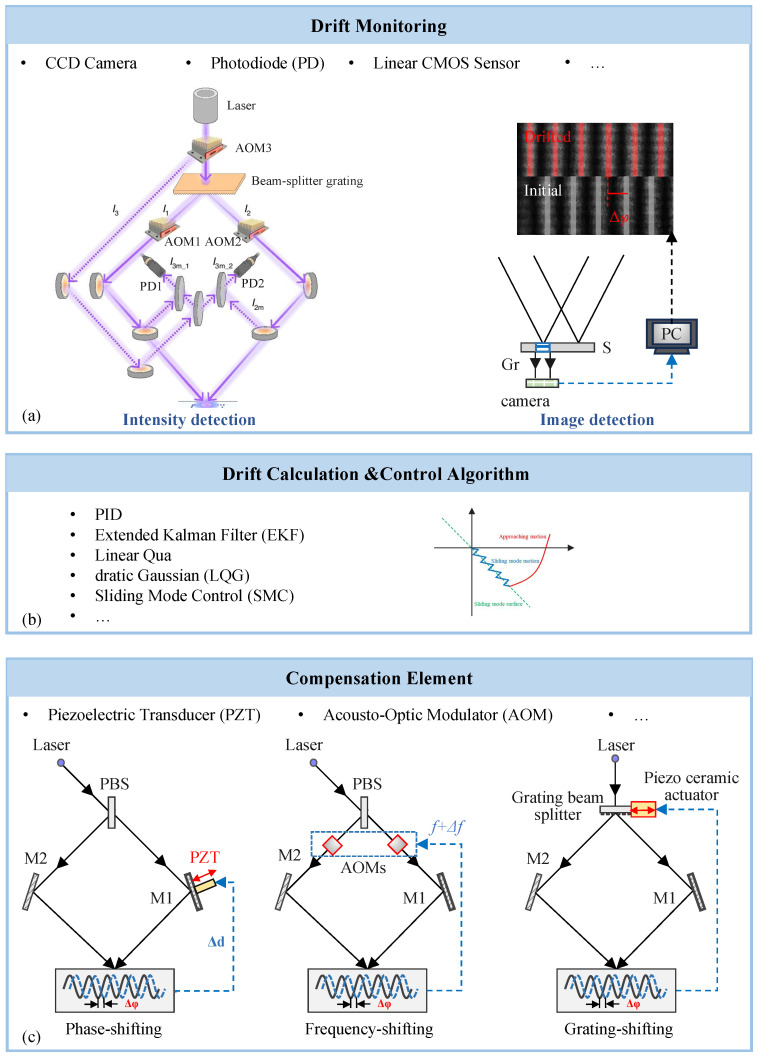
Schematic overview of active fringe-locking techniques in interference lithography. (**a**) Drift monitoring methods, including intensity-based and imaging-based methods. Reprinted from [155]. (**b**) Drift calculation and control algorithms. (**c**) Compensation methods: phase-shifting, frequency-shifting, and grating-shifting.

**Active Fringe Stabilization Techniques.** Active stabilization systems dynamically compensate for phase fluctuations in interference fields during exposure via real-time monitoring and closed-loop adjustment through optical compensators, effectively keeping interference fringes stationary or stable throughout the lithography process [203,204]. Such systems rely on highly sensitive phase drift detection, typically achieved via intensity-based or imaging-based methods, with commonly used sensors including photodiodes and cameras (e.g., CCD, CMOS, EMCCD), which is illustrated in Figure 9. In large-area interference lithography systems where the exposure field is expanded, a reference grating is often inserted into the interference region to generate a reference fringe pattern, thereby magnifying phase drift signals for observation and control [205,206,207]. Alternatively, self-referenced fringe generation from the latent image grating has also been demonstrated [208,209,210]; however, the resulting fringe intensity is weak, necessitating the use of ultra-sensitive imaging detectors.

In scanning interference lithography systems, where beams are not expanded, inserting a reference grating into the interference region is impractical. Instead, a portion of each beam is extracted via beam splitting and interfered in an auxiliary optical path, where phase drift is translated into intensity variation and monitored using photodetectors [211,212,213,214].

Depending on the compensation mechanism, active fringe stabilization can be categorized into three main types, and the Table 2 indicates their characteristics:

Phase-shifting stabilization: This method compensates for fringe drift by modulating the optical path difference between interfering beams. Piezoelectric transducers (PZTs) are typically used to drive mirrors and precisely adjust the optical delay [215,216].

Frequency-shifting stabilization: Acoustic-optic modulators (AOMs) introduce a controlled frequency offset between beams, resulting in fringe motion at a known rate, which can then be stabilized via closed-loop feedback [217,218,219].

Grating-shifting stabilization: This approach adjusts the phase of interference by moving beam-splitting gratings within the optical path, thereby modulating the optical path difference between the split beams [220].

The *Stabilock II* system developed by Odhner Holographics exemplifies a commercial implementation, achieving phase compensation with a precision of 0.05λ using photodetector feedback and PZT actuation. Preston Young et al. employed CCD imaging and Fourier analysis to monitor fringe peak movement, realizing fringe stabilization with precision better than 1/100 of a fringe period. XU Yuan et al. proposed a dual-frequency heterodyne technique using a reference holographic optical element (HOE) for wide-range phase drift compensation with millisecond-level response time [221].

The responsiveness and stabilization accuracy of active locking systems are fundamentally limited by both the physical characteristics of actuators (e.g., PZT, AOM) and the design of control algorithms. High-performance actuators must exhibit sub-nanometer resolution and high-frequency response. In addition to traditional PID control [222], advanced control strategies such as Extended Kalman Filter (EKF) [223], Linear Quadratic Gaussian (LQG) [224], and Sliding Mode Control (SMC) [225] have been introduced to enhance robustness and adaptability under multi-source disturbance and parameter uncertainties.

Active fringe and phase stabilization reduces sensitivity to thermal drift and mechanical vibrations, ensuring high pattern fidelity. Nevertheless, real-time monitoring and feedback systems introduce additional cost and operational demands. Large-area exposures are particularly vulnerable to cumulative drift, highlighting the need for modular, automated stabilization approaches to achieve scalable, high-throughput manufacturing.

In summary, active fringe stabilization has become a cornerstone technology in high-precision interference lithography. It provides essential support for real-time fringe stabilization, particularly in scenarios requiring long-duration exposure or multi-region stitching, where system alignment and pattern fidelity are critical [226,227,228,229,230].

## 5. Conclusions and Outlook

### 5.1. Conclusions

As a core technique for the large-area, ultra-high-precision fabrication of periodic micro- and nanostructures, interference lithography fundamentally relies on the systematic control of interference fringe formation mechanisms, morphology modulation strategies, and fringe stability maintenance. This review has focused on three major research directions: wavefront optimization, polarization modulation, and fringe locking techniques. We have systematically summarized recent advances and key technological pathways in each area, highlighting the ongoing evolution of interference lithography in meeting increasingly complex manufacturing demands and in enhancing both pattern accuracy and operational stability.

Firstly, in terms of wavefront modulation, researchers have progressed from traditional spherical wave configurations toward beam shaping schemes based on concave lenses, aspherical elements, and even freeform optics. These developments enable high-uniformity collimation and wavefront shaping of Gaussian beams. Such techniques not only significantly improve fringe uniformity but also open up new possibilities for interference lithography on curved substrates and non-planar devices, demonstrating excellent system compatibility and process scalability.

Secondly, in the domain of polarization control of the interference field, polarization has evolved from a secondary tuning parameter into a critical degree of freedom for spatial pattern design and performance optimization. From classic half-wave plate and polarizer configurations to integrated thin-film polarization devices and multi-beam coordinated polarization systems, a systematic control framework has emerged. This framework supports the construction of 2D and 3D structures, optimization of diffraction efficiency, and enhancement of optical functionalities, greatly expanding the applicability of interference lithography to complex structural and functional device fabrication.

Thirdly, in terms of fringe stabilization, the three major active locking strategies—phase-shifting, frequency-shifting, and grating-shifting—have become increasingly mature. These approaches are now deeply integrated with high-sensitivity monitoring techniques (such as CCD imaging, photodetectors, and moiré fringe detection) and intelligent control algorithms (including extended Kalman filtering, linear quadratic Gaussian control, and sliding mode control). Together, they enable high-precision, low-latency compensation of fringe phase and period drift, making them particularly well-suited for stability-critical applications such as step-and-repeat stitching and long-duration exposures. These advances are propelling interference lithography toward greater intelligence and robustness.

However, it should be emphasized that wavefront modulation, polarization control, and fringe locking techniques still face notable limitations in practical implementation and industrial deployment. Wavefront optimization typically relies on high-quality aspherical elements or adaptive optics, which inevitably increase system cost and alignment complexity. Polarization modulation, while highly effective in enhancing fringe contrast and expanding structural design flexibility, often involves intricate design processes and imposes stringent requirements on structural configurations; moreover, the performance and applicability of associated optical components remain constrained, thereby limiting their long-term stability in high-throughput production environments. Fringe stabilization strategies substantially improve the robustness of interference lithography against environmental disturbances, yet they simultaneously elevate process thresholds and operational costs. Overall, although these approaches have proven indispensable in advancing performance, they also introduce significant complexity and financial burdens, meaning that techniques readily adopted in research settings often require careful trade-offs between precision, cost, and scalability when transitioning toward industrial-scale manufacturing.

Despite the significant progress of interference lithography in high-throughput, high-uniformity periodic structure fabrication, its broader adoption and application still face several technical challenges.

### 5.2. Challenges and Future Directions

**Limited pattern morphology complexity:** Current interference lithography (IL) techniques remain constrained in their ability to control the spatial dimensionality and degrees of freedom of patterned structures. Since interference fields inherently arise from the superposition of a limited number of coherent beams, they naturally favor the formation of regular periodic patterns, resulting in limited flexibility for non-periodic structure design. Although strategies such as spatial light modulators (SLMs), phase encoding, and polarization multiplexing have been explored to enhance programmability and design freedom, these approaches often face challenges related to system stability, modulation precision, and computational complexity. Consequently, their maturity for realizing large-area, scalable, non-periodic structures remains limited. This shortcoming has restricted IL’s potential in the fabrication of multifunctional sensors, where more complex structures are often required to enable multi-parameter detection (e.g., displacement, refractive index, wavelength, and strain) or broadband optical sensing [231,232]. In such cases, insufficient structural complexity can limit both sensitivity and selectivity.

To overcome these bottlenecks, several strategies may be envisioned. First, combining dynamic multi-beam control with real-time wavefront shaping—enabled by high-refresh-rate SLMs or digital micromirror arrays—can achieve spatiotemporal composite encoding of interference fields, thereby generating heterogeneous patterns with distinct functional zones in a single exposure. Second, joint phase–polarization multiplexing could enable parallel regulation of multiple interference subfields within a single optical setup, allowing complementary structures to be fabricated on the same substrate and thus realizing multimodal sensing functions. Third, hybrid lithography schemes that integrate IL with scanning or mask-based lithography could enable cross-scale fabrication of complex structures [233]. For sensor manufacturing, advancing pattern complexity is particularly critical, as purely periodic structures are inherently limited in sensing applications, whereas non-periodic or quasi-periodic architectures provide enhanced multifunctionality and superior performance for spectral and biochemical sensing [234,235,236].

**Lack of quantitative evaluation and parameter-performance mapping:** Most existing IL studies have focused on proposing process schemes and validating experimental feasibility, but they lack systematic and quantitative methodologies to evaluate the correlation between modulation parameters and final pattern performance. For example, metrics such as wavefront shaping uniformity, polarization matching, and fringe stabilization accuracy are often described in terms of single-parameter indicators like contrast or period error, which cannot be directly mapped to device-level performance metrics such as sensor sensitivity, resolution, or linearity. This absence of parameter–performance mapping has led to process optimization being largely experience-driven, lacking standardized benchmarks and limiting reproducibility across platforms and industrial adoption.

To address this limitation, it is essential to establish a standardized quantitative evaluation framework that integrates interference-field simulation, structural reconstruction, and performance modeling, thereby building a full-chain link between interference field modulation, structural features, and device performance. In addition, digital twin models and machine learning algorithms can be leveraged to construct nonlinear parameter–performance mappings from extensive simulation and experimental data, thus enabling inverse optimization of process design. This direction is particularly valuable for sensor applications. For instance, in displacement sensing, the correlation between fringe phase stability and detection resolution can be explicitly defined through standardized models, providing reliable guidance for developing sensors with sub-nanometer accuracy.

**System complexity and limited industrial scalability:** Representative IL systems, such as scanning-type exposure platforms, are typically large in size, optically complex, and costly while requiring high-level expertise for operation and maintenance. While such complexity may be manageable in laboratory environments, it significantly constrains scalability and cost-effectiveness in industrial adoption and large-scale sensor manufacturing. In particular, sensor fabrication often demands large-area, high-throughput, and batch-consistent patterning capabilities, which current IL systems struggle to deliver without sacrificing either precision or cost efficiency.

Future research may address this bottleneck along two main directions. On the one hand, the development of compact IL systems through the integration of freeform optics, miniaturized interferometric modules, and advanced optical integration can reduce reliance on bulky platforms and high-precision mechanical alignment. On the other hand, scalable process-expansion strategies with lower system complexity should be developed to enable efficient enlargement of processing areas while ensuring stability and structural consistency.

Ultimately, optimizing system complexity and scalability is of paramount importance for sensor manufacturing. High-quality, industry-adaptable IL processes not only ensure batch-to-batch reproducibility and device stability but also broaden the industrial application horizon of sensing technologies, paving the way for their deployment in next-generation high-performance and mass-manufactured sensor systems.

In summary, the future of interference lithography will evolve from being “precision-driven” to “function-driven,” with core scientific challenges shifting toward integrated inverse design and closed-loop optimization around the target pattern—encompassing the entire pattern–field–system framework. For example, in flexible electronics, IL enables scalable fabrication of nanostructures on polymer substrates for displays, sensors, and energy devices. IL-based periodic nanostructures also play a key role in biosensing and lab-on-chip platforms, where strong field confinement and large-area uniformity markedly improve detection sensitivity. These applications highlight the shift of IL toward function-driven nanofabrication [237]. By incorporating emerging technologies such as machine learning, digital twins, and intelligent control, data-driven modulation strategies and adaptive manufacturing architectures can be established. These advances will lay a solid foundation for the widespread application of interference lithography in next-generation optoelectronic devices, functional structural materials, and mass-production-grade micro/nano fabrication.

## Figures and Tables

**Table 1:** Comparison of interference lithography techniques.

Reference	Interference Structure	Min. Period	Uniformity	Exposure Throughput
[102]	Orthogonal Two-Axis Lloyd’s Mirror	500 nm	High uniformity	Exposure area limited by the aperture of the holographic lens
[103]	Orthogonal Two-Axis Lloyd’s Mirror	720 nm	High uniformity	Single-shot exposure of 30 × 30 mm^2^
[104]	Non-Orthogonal Two-Axis Lloyd’s Mirror	1000 nm	/	Single-shot exposure of 100 × 100 mm^2^
[84]	Single-Axis Lloyd’s Mirror	1000 nm	Thickness non-uniformity: 5%	Exposure of 25 mm diameter area; Exposure time: 30 min
[105]	Three-Beam Interference	365 nm	Etched hole size std: <3%	Limited only by sample stage speed
[88]	EUV Interference	14 nm	Low uniformity	Low
[93]	Plasmonic Interference	22 nm	/	Theoretical high

**Table 2:** Comparison of active fringe stabilization techniques.

Technical Types	Phase-Shifting	Grating-Shifting	Frequency-Shifting
**Controlled Degrees of Freedom**	Simultaneous phase and period	Phase only	Primarily phase
**Actuator**	PZT linear actuator and PZT rotation stage	PZT linear actuator and beam splitter grating	Acousto-Optic Modulator (AOM)
**Advantages**	Simple, direct phase control	Insensitive to downstream optics	High-speed response, unlimited phase control range
**Disadvantages**	Bandwidth limited	Mechanical actuator limits bandwidth; single-axis	Highest system complexity and cost; nonlinear response
**Control Bandwidth**	>250 Hz	>500 Hz	>800 Hz (up to 2.5 kHz with lead controller)
**Control Accuracy**	RMS: Phase drift < 9.0 × $10^{-3}$ periods; Period drift < 1.5 × $10^{-3}$ periods	3σ: 0.13 rad (±0.021 periods)	3σ: <0.0693 rad (±0.01 periods)

## Appendix A. Comparison of Micro/Nano-Patterning Technologies

**Table A1:** Comparison of representative micro/nano-patterning technologies.

Technology	Resolution Limit	Throughput	Scalability	Design Flexibility
Interference Lithography (IL)	∼20 nm (λ/2n)	High	Wafer-scale	Medium (periodic patterns)
Direct Laser Interference Patterning (DLIP)	∼λ/2 (material dependent)	Very high	m^2^-scale (roll-to-roll possible)	Medium (periodic, tunable)
Laser-Induced Periodic Surface Structures (LIPSS)	∼λ/2 to λ (plasmonic effect)	High (scanning-based)	Large-area with beam scanning	Low–medium (self-organized)
Electron-Beam Lithography (EBL)	<5 nm	Low	Small-scale	High (arbitrary patterns)
Laser Direct Writing (DLW)	∼100 nm	Medium	Moderate	High (arbitrary patterns)
Nanoimprint Lithography (NIL)	∼10 nm (template dependent)	High	Wafer-scale	Medium (template dependent)
Photolithography	<10 nm (with EUV)	Very high	Wafer-scale	High (arbitrary patterns)
Focused Ion Beam (FIB)	∼5 nm	Low	Small-scale	High (arbitrary patterns)
Self-Assembly Methods	∼10 nm (block copolymer limit)	High	Wafer-scale	Low (limited control)
**Technology**	**Surface Compatibility**	**Cost-Effectiveness**		**Typical Applications**
Interference Lithography (IL)	Broad (various substrates)	High (for periodic patterns)		Photonic crystals, energy devices, sensors
Direct Laser Interference Patterning (DLIP)	Excellent (metals, polymers, ceramics, glass)	Very high		Tribology, wettability control, thin film functionalization
Laser-Induced Periodic Surface Structures (LIPSS)	Excellent (metals, dielectrics, semiconductors)	High		Biomimetic surfaces, plasmonics, wettability, anti-reflection
Electron-Beam Lithography (EBL)	Wide (conductive coating often needed)	Low		Mask fabrication, prototype development
Laser Direct Writing (DLW)	Good (polymers, resists)	Medium		Microstructure writing, rapid prototyping
Nanoimprint Lithography (NIL)	Good (various surfaces)	High		Mass replication, functional surfaces
Photolithography	Excellent (well-developed)	Medium–low (equipment cost)		Semiconductor production, microelectronics
Focused Ion Beam (FIB)	Limited (damage sensitive)	Low		Localized micromachining, mask repair
Self-Assembly Methods	Good (surface chemistry dependent)	Very high (low cost, low precision)		Low-cost templates, initial pattern formation
